# Effect of Minimal Individual or Group Enhancement in an eHealth Program on Mental Health, Health Behavior, and Work Ability in Employees With Obesity: Randomized Controlled Trial

**DOI:** 10.2196/66518

**Published:** 2025-07-07

**Authors:** Siniriikka A Männistö, Joona Muotka, Laura-Unnukka Suojanen, Raimo Lappalainen, Kirsi H Pietiläinen, Riitta Korpela

**Affiliations:** 1 HealthyWeightHub, Endocrinology, Abdominal Center, Helsinki University Hospital and University of Helsinki, Helsinki, Finland; 2 Obesity Research Unit, Research Program for Clinical and Molecular Metabolism, Faculty of Medicine, University of Helsinki, Haartmaninkatu 8, Helsinki, 00014, Finland, 358 442351020; 3 Department of Psychology, University of Jyväskylä, Jyväskylä, Finland; 4 Medical Nutrition Physiology, Department of Pharmacology and Research Programme for Human Microbiome, Faculty of Medicine, University of Helsinki, Helsinki, Finland

**Keywords:** eHealth, mental health, health behavior, obesity, obese, weight, acceptance and commitment therapy, healthy weight coaching, coach, coaching, ACT, psychotherapy, RCT, randomized, controlled trial, occupational health, burnout, eating habits, weight management, health intervention, virtual support, randomized controlled trial

## Abstract

**Background:**

Mental health problems and adverse health behaviors are enriched in individuals with obesity and need to be considered in weight loss interventions. Regarding weight loss, hybrid interventions combining digital and in-person elements have proven superior to eHealth-only interventions. However, it remains unclear whether minimal group or individual enhancement could bring additional benefits to the mental health and health behavior domains in individuals with obesity.

**Objective:**

This study aimed to explore whether minimal group or individual enhancements could offer additional benefits to an eHealth intervention in relation to mental health, perceived work ability, and health behavior in a sample of occupational health patients with obesity. In addition, the study sought to examine the overall effects of the health behavior–focused intervention across these domains.

**Methods:**

This study was a randomized controlled trial with a 12-month intervention (March 2021-2022), with selected variables followed for another 12 months without additional support. Recruited from occupational health care, 111 working-age adults with BMI 30‐40 kg/m^2^ were randomized to one of the 3 treatment arms, such as eHealth, eHealth+Group, or eHealth+Individual. All treatment arms received a web-administered, coach-assisted eHealth program based on acceptance and commitment therapy, and, in addition, the eHealth+Group and eHealth+Individual arms received 3 remotely facilitated group or individual meetings. The overall intervention effects were evaluated quasi-experimentally by combining the 3 treatment arms into a single eHealth arm. Participants were assessed for depression (the Beck Depression Index 21 [BDI-21]), burnout (Bergen Burnout Inventory 18 [BBI-15]), perceived work ability, eating behavior (ecSatter 2.0, Three Factor Eating Questionnaire [TFEQ], and Binge Eating Scale [BES]), and physical activity (Baecke Habitual Physical Activity Questionnaire, BHPAQ).

**Results:**

We observed inconsistent fluctuations between the treatment arms in depression and burnout scores, indicating a lack of meaningful intervention effects despite statistical significance. Therefore, none of the treatment arms showed superiority over another. Across all participants, depression showed an estimated mean decrease of 2.5 BDI-21 points, with older participants experiencing a greater reduction in depressive symptoms. Furthermore, binge eating tendency decreased by 4.9 BES points during the 12-month intervention. We also observed increases in eating competence, controlled restraint, and physical activity. However, the 24-month measurements showed an adverse effect on eating competence, especially on attitudes toward eating and food, during the follow-up period without further support.

**Conclusions:**

Minimal enhancement through either group or individual video-conference meetings did not provide additional benefits in the mental health or eating habit domains compared with the eHealth intervention alone. Nevertheless, our results indicate that eHealth interventions for weight loss have the potential to reduce depression symptoms and binge-eating tendencies, while also improving eating competence and physical activity across the study population. Continued support may be necessary to sustain positive changes.

## Introduction

Obesity and mental health problems are intricately connected, with evidence suggesting a bidirectional relationship between the two [1,2]. Depression, one of the most extensively studied mental health issues, increases the risk of obesity by 58%, while obesity elevates the risk of depression by 55% [3]. Furthermore, both obesity and depression are significant risk factors for reduced work ability [4,5], and occupational burnout has been linked to these conditions [6-8]. Several mechanisms, including weight-related stigma [9], hypothalamic-pituitary-adrenal axis dysregulation [10], and adverse health behaviors [11], have been proposed to explain these associations, with these factors likely interacting in complex ways [12].

Originally developed for mental health treatment, transdiagnostic treatment approaches target common underlying factors across multiple disorders [13] and are also applied in obesity treatment to support weight management [14]. Among these, cognitive behavior therapy is often considered the first-line treatment option. However, research suggests that cognitive behavior therapy may not consistently produce desired weight loss outcomes, and its emphasis on thought suppression could even contribute to binge eating and weight gain [15,16]. As a result, mindfulness-based methods have gained popularity as alternatives in psychological obesity treatment, with acceptance and commitment therapy (ACT) showing the most consistent results in reducing BMI in adults with overweight and obesity [17].

ACT aims to enhance psychological flexibility, a trait linked to improved psychological well-being [18,19], and has demonstrated efficacy not only in weight management but also in the treatment of various mental and somatic health conditions [20]. This is particularly relevant in obesity treatment, as individuals with obesity face an elevated risk of mental health issues and eating disorders, which can be exacerbated by weight-normative interventions that emphasize weight loss [21,22]. In their review, Iturbe et al [21] found that while ACT supports psychological well-being even in weight loss contexts, more favorable outcomes were observed in interventions that prioritized health promotion over weight reduction. Consequently, ACT-based interventions that focus on sustainable health behaviors rather than weight loss alone may be more effective in promoting long-term well-being.

Furthermore, ACT is applicable in a variety of formats [23], including digital and hybrid interventions [24,25]. These modes of delivery are underscored by the widespread prevalence of obesity and mental health issues, as they have shown great potential for cost-effectiveness, scalability, and accessibility [26,27]. Hybrid interventions, which integrate digital and in-person elements, have shown superior outcomes in somatic metrics compared with fully digital approaches [28,29]. Nevertheless, the introduction of in-person elements reduces the scalability of the intervention, emphasizing the need for minimal face-to-face input.

To date, it remains unclear whether group or individual contact could bring additional benefits to mental health and health behavior domains following a weight-management intervention, and if these benefits are notable following minimal face-to-face enhancement. To take a step forward in evaluating the optimal mode and frequency of contact for digital support [30], we aimed to explore whether a 3-time group or individual enhancement could offer additional benefits to a coach-assisted eHealth intervention in relation to mental health, perceived work ability, and health behavior. In addition, we examined the overall effects of the health behavior–focused intervention that combined ACT and behavioral components across these domains. We hypothesized that the hybrid treatment arms would be more effective than the eHealth-only arm. Given the mixed findings of previous research, we could not predict whether the group or individual format would differ in overall effectiveness.

## Methods

### Study Design

This study was a randomized controlled trial (RCT) aiming to improve participants’ health behavior and thus, weight loss [31]. Participants were assigned to one of the 3 treatment arms: eHealth, eHealth+Group, or eHealth+Individual. Each treatment arm used an eHealth program, Healthy Weight Coaching (HWC), extensively detailed by Suojanen et al [32]. In addition, the hybrid treatment arms received 3 remotely facilitated group or individual sessions. In all 3 treatment arms, the intervention lasted 12 months (March 2021-2022), followed by a 12-month unsupported follow-up period.

The HWC integrates behavioral weight management themes (eg, eating habits, physical activity, sleep, and stress management) with the core processes of ACT (values, committed action, contact with the present moment, acceptance, defusion, and self as context) with information and exercises covering these themes (refer to Table S1 in Multimedia Appendix 1 for a more detailed description of the intervention). The aim of the HWC is to teach weight-management skills and improve psychological flexibility, resulting in long-term weight loss. Participants were asked to complete 1 “session” per week, covering information and exercises. They received tailored one-on-one written feedback every 2 weeks for the initial 3 months and every 3 weeks for the remaining year, totaling 19 interactions. The same coach was responsible for the HWC program support and any supplementary contacts. All 3 coaches had master’s degrees in nutrition and further education in psychology and were randomly allocated to one-third of the participants in each treatment arm.

Helsinki city employees with a BMI of 30-40 kg/m^2^ were recruited for the study from February to March 2021 via advertisements on city employees’ platforms and the nursing staff of Occupational Health Care Helsinki. Besides BMI, inclusion criteria included the capacity to engage in potential group or individual sessions and access to a computer with internet connectivity. Exclusion criteria included participation in other weight loss programs, recent pregnancy or lactation, weight fluctuations of more than 5 kg in the past 3 months, major medical conditions impacting safety or readiness for weight loss, and the use of weight loss medication.

After passing the initial screening by a research assistant, eligible candidates underwent phone-based health screening conducted by a medical doctor to assess their health status and medications. Suitable participants then proceeded with laboratory assessments and body composition measurements. When written consent was provided, the statistician (JM) used stratified random allocation for SPSS (IBMCorp) to randomize the participants into 3 treatment arms stratified by sex, age, and BMI. The sample size was determined with a priori power analysis by a statistician (JM) and was based on the expected weight change.

### Treatment Arms

#### eHealth

Participants received the HWC eHealth program, which included a 20-minute kickoff call 3 weeks into the program to monitor their progress. They also received written feedback every 2 weeks for the first 3 months and then every 3 weeks for the rest of the year, totaling 19 contacts.

#### eHealth+Group

Participants received the HWC eHealth program (as detailed in item 1) and participated in 3 remotely facilitated group meetings via video (each lasting 2 h). Due to the COVID-19 pandemic, these meetings were held remotely and included brief introductions to topics, such as goal setting, stress management, and self-compassion, followed by small-group discussions (Table S2 in Multimedia Appendix 1). Group meetings were held at 1, 6, and 10 months during the 12-month treatment period.

#### eHealth+Individual

Participants received the HWC eHealth program (as described in item 1) and engaged in 3 remotely facilitated individual meetings via video (each lasting 45 min). These sessions were tailored to each participant’s current needs, recognizing their achievements and addressing challenges. Individual meetings were held at 1, 6, and 10 months within the 12-month treatment period.

### Outcome Measures

#### Depression Symptoms

Depression symptoms were measured using the Beck Depression Index 21 (BDI-21) [33]. BDI-21 is one of the most used depression indicators measuring both cognitive and somatic symptoms of depression [34,35]. Question 19 was modified to ask if the participant had lost or gained weight. Since all participants were involved in a weight-management intervention, weight loss was considered intentional and was scored as zero. Each question is scored on a scale of 0‐3. The total score of the questionnaire ranges from 0 to 63, with higher scores indicating more severe symptoms. A score of 0‐12 indicates a normal condition, 13‐18 indicates mild depression, 19‐29 indicates moderate depression, and 30 or above indicates severe depression.

#### Burnout Symptoms

Burnout symptoms were measured using the Finnish version of the Bergen Burnout Inventory (BBI-15) [36]. The BBI-15 assesses exhaustion, cynicism, and reduced professional efficacy, which are the 3 key symptoms of burnout according to the most widely accepted definition [37]. Each question is scored on a scale of 1‐6, with a total score range of 15‐90. Higher scores indicate more severe symptoms. The total score of the questionnaire is calculated as the sum of these subcategories.

#### Perceived Work Ability

Perceived work ability was measured using a subjective single-question assessment (“What score would you give your current work ability on a scale of 0‐10?”), with 8‐10 indicating good, 6‐7 reduced, and 0‐5 poor work ability. This question is part of the work ability index and has a good predictive value for work ability risk [38,39].

#### Eating Behavior

Eating behavior was measured with 3 different questionnaires covering different aspects of eating behaviors and attitudes. Eating competence was measured with the Finnish version of the ecSatter Inventory 2.0 (ecSI2.0) questionnaire [40,41], including 4 subcategories: eating attitudes (has positive attitude toward food), food acceptance (is comfortable with familiar foods and skilled at trying unfamiliar foods), internal regulation skills (recognizing and trusting hunger and satiety signals in choosing food amounts), and contextual skills (prioritizing meals and having the skills and resources for food handling). All questions are scored on a scale of 0‐3, with a total score range of 0‐48. Higher scores indicate better eating competence, with a cutoff score of 32 used to indicate good eating competence. Cognitive restraint, uncontrolled eating, and emotional eating were measured with an 18-item Finnish version of the Three Factor Eating Questionnaire (TFEQ-R18) [42,43], reporting the relative proportion (0%‐100%) of the maximum possible raw score for each subscale (the higher the value, the greater the level). Binge eating was measured by the Binge Eating Scale (BES) [44,45]. The BES has 16 items scored 0‐2 or 0‐3, with the total score range of 0‐46. A score below 20 is considered normal, whereas scoring 20‐29 indicates moderate binge eating tendency, and 30 or above indicates a severe binge eating tendency.

#### Physical Activity

Physical activity was measured with the Baecke Habitual Physical Activity Questionnaire (BHPAQ), covering work, sports, and leisure [46]. The questionnaire has 16 items, each scored 1‐5. For each subcategory (work, sports, and leisure), a mean score is calculated, and the overall score is the total sum of the subcategory scores. A higher score indicates greater physical activity.

Participant assessments were conducted at baseline, midintervention (6 mo), postintervention (12 mo), and follow-up (24 mo). Baseline, midintervention, and postintervention questionnaires were answered as a part of the eHealth program, and follow-up questionnaires were mailed to all participants regardless of their activity in the program. Hence, most questionnaires cover data up to 12 months, while a selection of questionnaires also cover 24-month follow-up data.

Of the questionnaires, BDI-21, BBI-15, perceived work ability, TFEQ-R18, and BHPAQ were added to the HWC program at a specific time point (0, 6, and 12 mo), while the ecSI2.0 and the BES were integrated into the program structure. Since participants filled the ecSI2.0 and the BES while progressing through the program, not everyone reaching the recommended pace of 1 session per week, we pooled the answers to the 0-, 6-, and 12-month time points from the nearest actual answering time (baseline questionnaire answered during intervention months ranged 0‐3, 6-month questionnaire during months ranged 4‐8, and 12-month questionnaire during months ranged 9‐12). We report the subscales only for variables where the total score showed significance, except for the TFEQ, for which only subscale results are provided. Age, sex, and profession were retrieved from the electronic health record system, and BMI was calculated from height and weight measured by trained research assistants [31].

### Statistical Methods

Data analysis was conducted using IBM SPSS Statistics (version 28.0.0.0). Baseline differences were analyzed by Fisher exact test for categorical variables, ANOVA for the parametric continuous variables, and Kruskal-Wallis nonparametric continuous variables.

EcSI2.0 was normally distributed. Perceived work ability, ecSI’s subcategories Eating attitude and Internal regulation skills, BHPAQ, and its subcategory Leisure had negatively skewed distribution, and thus, inverted variables were used in the analysis with gamma distribution and log link function. All other variables had positively skewed distribution, and thus, gamma distribution with log link function was used in the analysis.

We used the Generalized Estimating Equations method [47] to analyze the differences between the treatment arms and overall change over time. The Generalized Estimating Equations method includes all available data points and uses estimated means in the analysis, applying the intention-to-treat principle to decrease attrition bias. Following the unadjusted primary analysis, we added sex, age, and weight change (0-6 mo and 0-12 mo) to the model as covariates to explore their impact on the results. For mental health variables, we also included psychotropic medication use or changes in the medication (0-12 mo and 12-24 mo) as covariates (Table S3 in Multimedia Appendix 1). Considering the model size, each covariate was initially tested individually in a preliminary covariate model, reported in Multimedia Appendix 1. Statistically significant covariates were then combined into a single model, and the results of the full covariate model were reported in the text. To avoid multicollinearity, the 2 time points of weight change and medication change variables were tested separately in different models.

Little’s missing completely at random (MCAR) analysis was used to determine whether the data were MCAR. With variables whose data were not MCAR, we used Full Information Maximum Likelihood estimation with Mplus computer software version 8.11 to correct for the potential bias. Crosstabs with Fisher exact test were used to analyze change in depression and burnout incidence between the measurement points. Incidence was separately calculated for completers and for the entire baseline sample with last observation carried forward. The Spearman correlation coefficient was used for correlation analysis, and the Dancey and Reidy definition was applied to determine correlation strength [48].

Cohen *d* effect size was calculated for the differences between different arms, using the square root of the average variance of measures and correcting for baseline differences. In the analyses for the entire group, the pooled SD between measurement times was used to calculate the effect size [49]. Effect size of 0.2 was considered small, 0.5 medium, and 0.8 large [50]. Figures were drawn with GraphPad Prism.

### Ethical Considerations

The study protocol was approved by the Ethics Committee of the Helsinki and Uusimaa Hospital District (29.4.2020, HUS/922/2020). Informed consent was obtained from all individuals participating in the study. To protect privacy and confidentiality, the data were pseudonymized and securely processed to prevent identification of individual participants. Participants received access to the intervention and their personal health data but did not receive any additional compensation. Detailed reporting of the study is provided in the CONSORT-EHEALTH checklist (Checklist 1) .

## Results

### Baseline Characteristics

**Figure 1.**
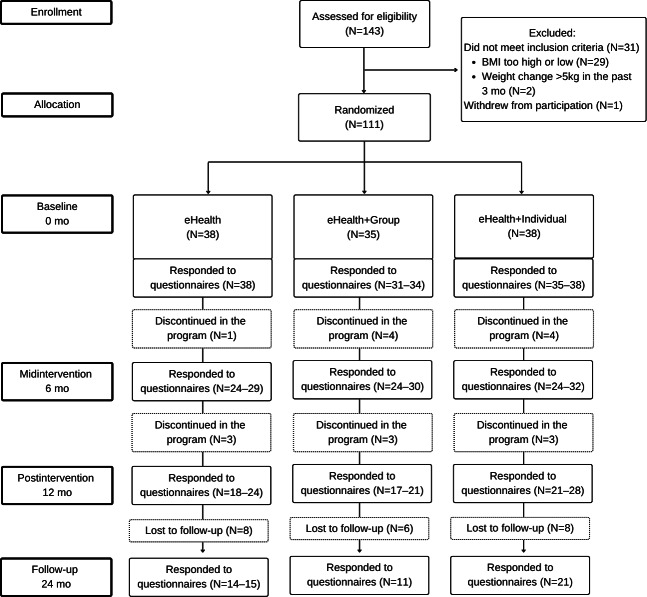
Flowchart of participant progress through the three study groups (eHealth, eHealth+Group*,* and eHealth+Individual) in the randomized controlled trial, with a total sample size of N=111.

After the screening, 111 participants fulfilling the inclusion criteria were randomized into the 3 treatment arms with a 1:1:1 ratio (Figure 1). The mean age was 50 (SD 8.9) years and mean BMI was 34.2 (SD 2.9) kg/m^2^ (Table S4 in Multimedia Appendix 1). Of the 111 participants, 90 (83%) were female and 97 (87%) were “white collar” workers (manager, specialized expert, expert, or office and customer service worker).

Figure 1 illustrates group allocation and participant retention over the 24-month follow-up, showing variation in the number of respondents across questionnaire time points. As 1 participant did not reply to the baseline questionnaires, the total data pool analyzed for the mental health and health variables included 110 participants (Table S4 in Multimedia Appendix 1). Baseline characteristics did not differ between the treatment arms except for 1 subscale (leisure) of the physical activity questionnaire, where the eHealth+Group treatment showed lower baseline values than the other 2 arms. Regarding mental health and work ability, 36/110 (33%) participants indicated at least mild depression, 32/100 (29%) participants at least mild burnout, 36/100 (33%) participants reduced or poor perceived work ability, and 15/100 (14%) participants reported using psychotropic medication (Table S3 in Multimedia Appendix 1). Considering eating behavior, 16/110 (15%) participants indicated at least moderate binge eating tendency, and 91 (88%) participants had “poor” eating competence. No between-groups differences were observed in these categories.

The correlation between depression and burnout scores was moderate at baseline (*r*=0.563, *P*<.001), indicating that depression and burnout symptoms occurred largely in the same participants. Furthermore, the participants whose BDI score indicated clinical depression at baseline had a higher baseline burnout score (*P*<.001), lower perceived work ability (*P*<.001), higher emotional eating score (*P*=.02), and higher binge eating tendency score (*P*<.001) than nondepressed participants (Table S5 in Multimedia Appendix 1). The participants whose BDI score indicated clinical burnout at baseline had a higher depression score (*P*<.001), lower perceived work ability (*P*<.001), and lower eating competence (*P*=.03) at baseline compared with those without burnout. The participants using psychotropic medication had higher baseline depression (*P*<.01), lower perceived work ability (*P*<.001), and higher binge eating tendency (*P*<.001) than nonmedication users.

### Comparison of the Treatment Arms

To compare the impact of the 3 treatment arms concerning mental health, perceived work ability, and health behavior variables, we analyzed the interaction of the intervention arms over time (Table 1). Below we report results using the full dataset with estimated mean values to account for missing data, while completer results are presented in Table S6 in Multimedia Appendix 1.

**Table 1.** Estimated mean values (standard error) of mental health, perceived work ability, eating behavior, and physical activity variables at baseline, midintervention, postintervention, and follow-up, across treatment arms and all participants (N=110), analyzed using generalized estimating equations.

Variables	Baseline (0 mo), mean (SE)	Midintervention (6 mo), mean (SE)	Postintervention (12 mo), mean (SE)	Follow-up (24 mo), mean (SE)	Overall change (0-6-12[-24] mo)	
					Wald chi-square (*df*)	*P* value
Depression	10.13 (0.69)	9.08 (0.67)	7.62 (0.75)	—^a^	12.41 (1)	.002
eHealth	10.11 (1.08)	9.92 (1.24)	6.32 (1.07)	—	17.99 (4)	.001
eHealth+ Group	8.65 (1.07)	9.20 (1.05)	7.20 (1.20)	—	17.99 (4)	.001
eHealth+ Individual	11.47 (1.34)	8.18 (1.20)	8.76 (1.41)	—	17.99 (4)	.001
Burnout	39.68 (1.34)	43.75 (1.50)	40.53 (1.53)	42.75 (2.05)	10.47 (3)	.01
eHealth	40.84 (2.12)	46.24 (2.71)	42.09 (2.24)	41.89 (3.30)	12.91 (6)	.04
eHealth+ Group	37.50 (2.37)	44.84 (2.64)	36.48 (3.21)	43.12 (3.69)	12.91 (6)	.04
eHealth+ Individual	40.47 (2.43)	40.38 (2.40)	42.25 (2.36)	42.77 (3.34)	12.91 (6)	.04
Exhaustion	14.02 (0.48)	15.29 (0.63)	13.92 (0.57)	14.71 (0.71)	10.03 (3)	.02
eHealth	14.50 (0.70)	15.50 (0.85)	13.70 (0.73)	14.09 (1.23)	7.88 (6)	.25
eHealth+ Group	13.78 (0.96)	16.42 (1.39)	13.91 (1.22)	15.31 (1.22)	7.88 (6)	.25
eHealth+ Individual	13.71 (0.80)	14.01 (0.93)	13.92 (0.92)	14.66 (1.13)	7.88 (6)	.25
Cynicism	12.09 (0.49)	13.77 (0.57)	12.67 (0.60)	13.19 (0.74)	12.70 (3)	.005
eHealth	12.18 (0.79)	14.83 (1.12)	13.00 (1.00)	12.67 (1.14)	17.01 (6)	009
eHealth+ Group	11.44 (0.83)	14.28 (0.99)	11.63 (1.16)	13.74 (1.45)	17.01 (6)	.009
eHealth+ Individual	12.58 (0.90)	12.34 (0.80)	13.15 (0.96)	13.07 (1.17)	17.01 (6)	.009
Efficacy	13.93 (0.56)	15.15 (0.58)	14.17 (0.63)	14.98 (0.87)	5.87 (3)	.12
eHealth	14.16 (0.95)	15.93 (1.03)	15.35 (1.07)	15.01 (1.44)	10.83 (6)	.09
eHealth+ Group	13.38 (0.99)	15.59 (1.03)	11.74 (1.28)	14.68 (1.81)	10.83 (6)	.09
eHealth+ Individual	14.18 (0.99)	14.05 (0.97)	15.20 (0.88)	15.01 (1.34)	10.83 (6)	.09
Perceived work ability	7.86 (0.15)	7.50 (0.18)	7.80 (0.19)	7.67 (0.23)	5.19 (3)	.16
eHealth	8.00 (0.23)	7.56 (0.31)	8.29 (0.16)	7.69 (0.46)	9.02 (6)	.17
eHealth+ Group	7.76 (0.24)	7.62 (0.27)	7.95 (0.28)	7.59 (0.55)	9.02 (6)	.17
eHealth+ Individual	7.82 (0.31)	7.33 (0.34)	7.27 (0.41)	7.70 (0.27)	9.02 (6)	.17
Eating comp.	23.04 (0.76)	24.80 (0.87)	26.99 (0.91)	20.51 (0.95)	72.79 (3)	<.001
eHealth	23.97 (1.31)	22.55 (1.44)	27.34 (1.65)	23.06 (1.57)	6.46 (6)	.37
eHealth+ Group	22.20 (1.18)	23.24 (1.29)	26.90 (1.55)	19.79 (1.56)	6.46 (6)	.37
eHealth+ Individual	22.77 (1.40)	25.51 (1.73)	26.75 (1.50)	19.10 (1.53)	6.46 (6)	.37
Eating attitudes	8.82 (0.32)	8.40 (0.37)	9.38 (0.35)	6.79 (0.38)	44.81 (3)	<.001
Food acceptance	3.72 (0.22)	3.93 (0.23)	5.72 (0.26)	3.82 (0.31)	47.53 (3)	<.001
Regulation of food intake	4.87 (0.23)	5.26 (0.24)	4.30 (0.30)	4.92 (0.34)	9.55 (3)	.02
Eating context	5.66 (0.31)	7.25 (0.34)	7.70 (0.34)	5.41 (0.37)	51.27 (3)	<.001
Controlled restraint	38.91 (1.67)	51.59 (1.93)	46.70 (2.10)	—	45.65 (1)	<.001
eHealth	43.78 (3.04)	53.80 (4.05)	51.43 (3.92)	—	6.43 (4)	.17
eHealth+Group	37.8 (2.87)	47.39 (2.92)	42.48 (3.09)	—	6.43 (4)	.17
eHealth+Indiv	42.48 (3.09)	53.51 (3.02)	46.31 (3.49)	—	6.43 (4)	.17
Uncontrolled eating	44.44 (1.82	34.36 (1.99)	32.50 (2.23)	—	50.34 (1)	<.001
eHealth	45.07 (3.01)	36.09 (3.52)	32.37 (3.26)	—	2.79 (4)	.59
eHealth+Group	44.99 (3.11)	33.33 (3.44)	28.95 (3.34)		2.79 (4)	.59
eHealth+Indiv.	43.28 (3.32)	33.69 (3.31)	35.14 (4.31)	—	2.79 (4)	.59
Emotional eating	50.73 (2.39)	46.98 (2.35)	46.75 (2.90)	—	2.73 (1)	.26
eHealth	52.94 (4.36)	47.38 (4.80)	40.87 (3.81)	—	3.77 (4)	.44
eHealth+Group	49.06 (4.75)	44.57 (3.69)	48.10 (4.82)	—	3.77 (4)	.44
eHealth+Indiv.	49.73 (3.23)	48.90 (3.71)	50.36 (5.01)	—	3.77 (4)	.44
Binge eating	12.84 (0.71)	8.75 (0.74)	7.92 (0.86)	—	43.22 (1)	<.001
eHealth	13.18 (1.28)	8.76 (1.32)	8.09 (1.53)	—	0.41 (4)	.98
eHealth+ Group	12.72 (1.06)	8.39 (1.18)	7.48 (1.60)	—	0.41 (4)	.98
eHealth+ Individual	12.63 (1.29)	9.07 (1.32)	8.19 (1.21)	—	0.41 (4)	.98
Physical activity	10.86 (0.15)	11.09 (0.14)	11.39 (0.16)	—	13.50 (1)	<.001
eHealth	11.07 (0.24)	11.06 (0.23)	11.57 (0.27)	—	5.41 (4)	.25
eHealth+ Group	10.59 (0.21)	10.88 (0.22)	11.00 (0.23)	—	5.41 (4)	.25
eHealth+ Individual	10.90 (0.32)	11.30 (0.25)	11.53 (0.29)	—	5.41 (4)	.25
Work	2.12 (0.39)	2.18 (0.40)	2.15 (0.04)	—	7.62 (1)	.02
Sports	2.55 (0.10)	2.84 (0.09)	2.95 (0.10)	—	18.54 (1)	<.001
Leisure	6.19 (0.10)	6.07 (0.10)	6.29 (0.11)	—	7.31 (1)	.03

a Not applicable

**Figure 2.**
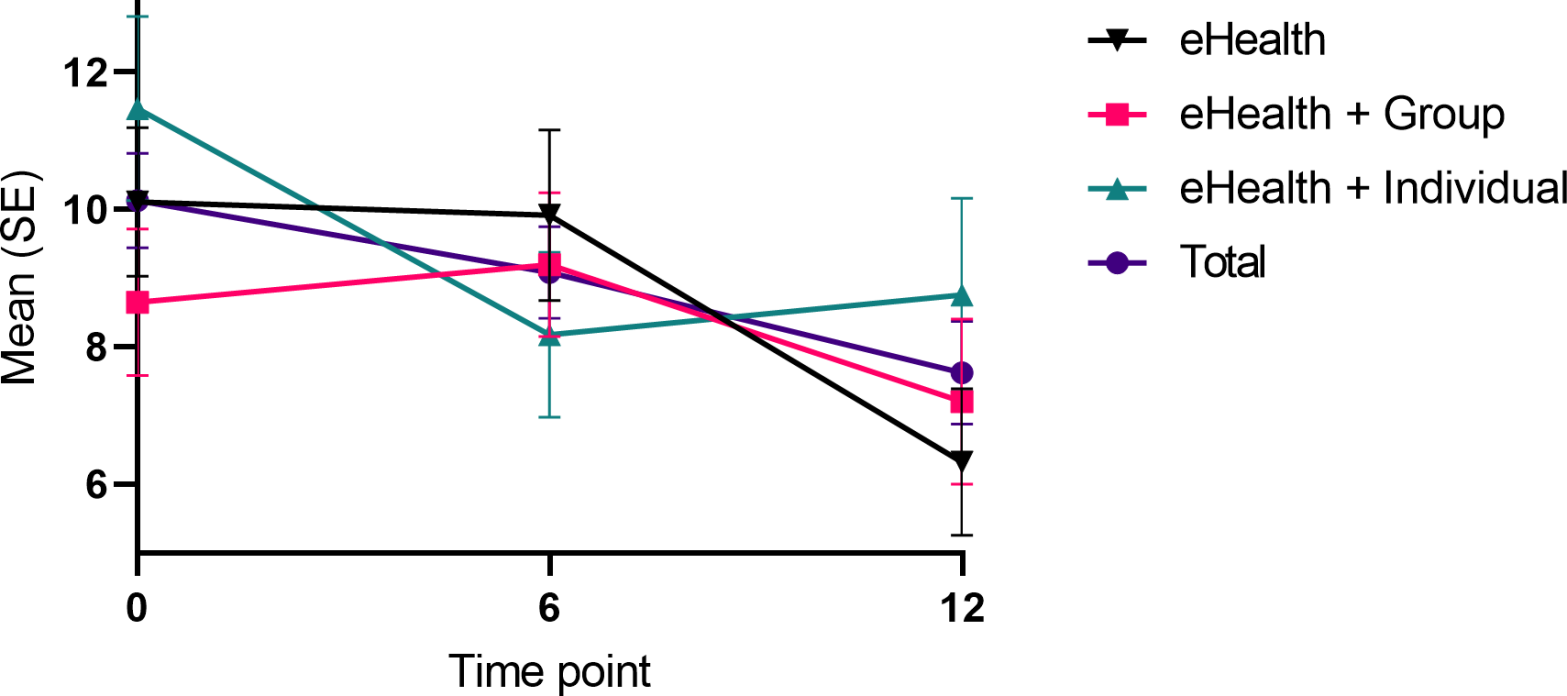
Changes in depression symptoms between the eHealth, eHealth + Group and eHealth + Individual treatment arms during the 12-month intervention.

In Table 1, the results for all participants are presented in the title row, while between-group results are reported in the “eHealth” rows. Concerning depression, we observed an overall time–group interaction (*χ*^2^_4_=17.99; *P*<.01), indicating that the treatment arms responded differently to the intervention (Table 1 and Figure 2). The significant differences between the groups were pinpointed between 0 and 6 months (*χ*^2^_1_=11.17; *P*<.01), and 6 and 12-month time points (*χ*^2^_1_=9.12; *P=*.01). Post hoc analysis comparing the 3 groups revealed that the eHealth+Individual yielded greater declines in the depression score compared with the eHealth (*χ*^2^_1_=6.73; *P*<.01; *d*=0.42) between 0- and 6-month time points. Between 6- and 12-month time points, the eHealth condition showed significantly greater decline in the depression score than the eHealth+ Individual condition (*χ*^2^_1_=8.87; *P*=<.01, *d*=0.14). The statistically significant time–group interaction was not observed between 0- and 12-month time points (*χ*^2^_1_=3.27; *P*=.20*)*, suggesting an absence of a relevant intervention effect. The covariate model showed that the between-group interaction was attributed to 12-month weight change (*χ*^2^_6_=20.43; *P*<.01), baseline use of psychotropic medication (*χ*^2^_6_=70.04; *P*<.001) and 0‐12 month changes in psychotropic medication use (*χ*^2^=73.93; *P*<.001). In the full covariate model, the time-group interaction did not remain significant (*χ*^2^_4_=7.37; *P*=.12), suggesting that the observed changes were not attributable to differences between the intervention arms.

**Figure 3.**
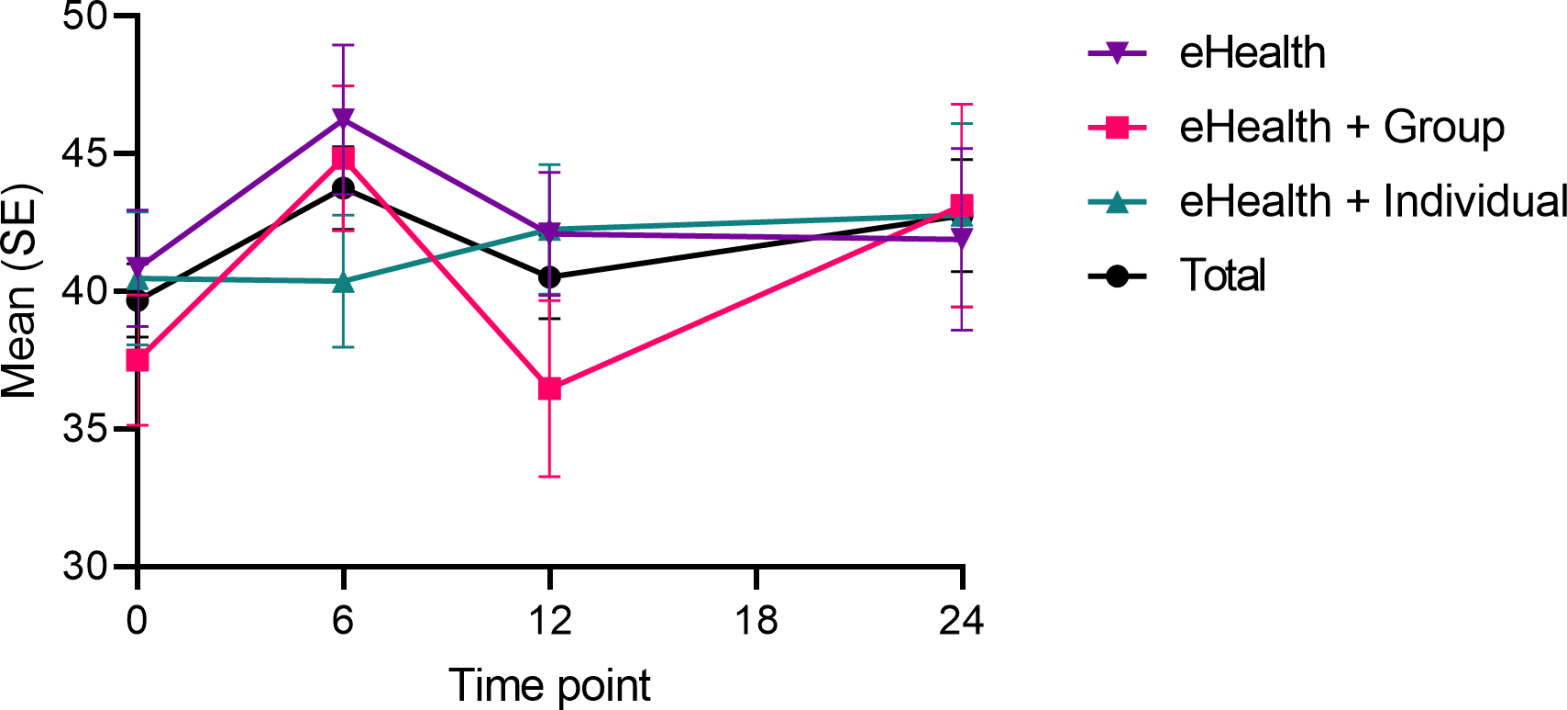
Changes in burnout symptoms between the eHealth, eHealth+ Group and eHealth+ Individual treatment arms during the 12-month intervention and further 12-month follow-up without additional support.

We also observed a statistically significant time–group interaction in burnout (*χ*^2^_2_=12.910; *P*=.04), pinpointed between the 0- and 6-month (*χ*^2^_2_=8.065; *P*=.02), and 6- and 12-month time points (χ^2^_2_=8.299; *P*=.02) (Table 1 and Figure 3). Between the 0- and 6-month time points, we observed no difference between the eHealth and the intervention arms. Between the 6- and 12-month time points, however, the eHealth+ Group treatment showed a greater decrease in burnout compared with the eHealth treatment (*χ*^2^_1_=4.611; *P=*.03*; d*=0.29). These differences were not statistically significant between 0- and 12-month (*χ*^2^_2_=0.73; *P*=.70*)* or 0- and 24-month time points (*χ*^2^_2_=3.60; *P*=.17*)*, indicating a lack of a meaningful intervention effect. Furthermore, the covariate model showed that the between-group interaction was driven by sex (*χ*^2^_8_=30.05; *P*<.001), baseline use of psychotropic medication (*χ*^2^_8_=80.35; *P*<.001) and 0‐12 month changes in psychotropic medication use (*χ*^2^_6_=70.38; *P*<.001). The time-group interaction did not remain significant (*χ*^2^_6_=9.75; *P=*.14) when using the full covariate model, indicating that none of the observed changes were attributable to differences between the intervention arms.

We did not observe significant time–group interactions in perceived work ability, eating behavior, or physical activity, indicating that the changes between the treatment arms did not differ over time (Table 1).

### Overall Effects

To study the overall impact of the intervention, we combined the 3 treatment arms into a single eHealth arm for further analysis. Across all participants and time points, we observed an estimated mean decrease of 2.5 BDI points in depression (*χ*^2^_1_=12.41; *P*<.01; *d*=0.38) (Table 1 and Figure 2). Covariate analysis revealed that older participants experienced a greater reduction in depressive symptoms (*χ*^2^_2_=8.74; *P*=.03). Notably, the main effect of time remained statistically significant in the full covariate model, controlling for age, and this significance was maintained when applying Full Information Maximum Likelihood estimation to correct for potential bias, both in the full model (*P*=.007) and age-controlled model (*P=*.02). Furthermore, we observed a statistically significant decrease in clinical depression incidence in the completer (*P=*.02) but not in the intention-to-treat analysis (*P=*.23; Tables S8a and S8b in Multimedia Appendix 1).

We found an increase in burnout symptoms between 0- and 6-month time points (*χ*^2^_1_=9.56; *P*<.01; *d*=−0.25), and a decrease between 6- and 12-month time points (*χ*^2^_1_=5.65; *P=*.02; Table 1). The results were attributed to changes in psychotropic medication at 0‐12 months (*χ*^2^_3_=20.08; *P*<.001), as the main effect did not remain significant (*χ*^2^_3_=5.55; *P*=.14) in the covariate model. The increase in the burnout score was not reflected on the clinical burnout incidence between the 0- and 6-month time points (Tables S9a and S9b in Multimedia Appendix 1). Perceived work ability did not change over time (Table 1).

Furthermore, we observed an increase in eating competence (*χ*^2^_3_=72.788; *P<.*001), controlled restraint (*χ*^2^_1_=45.65; *P*<.001), and physical activity (*χ*^2^_1_=13.501; *P*=.001, and a decrease in uncontrolled eating (*χ*^2^_1_=50.34; *P*=.001) and binge eating tendency (*χ*^2^_1_=43.22; *P*<.001; Table 1) during the 12-month intervention. However, the 24-month follow-up measurements showed an adverse effect in eating competence, especially in attitudes toward eating and food, in the absence of continued support.

**Figure 4.**
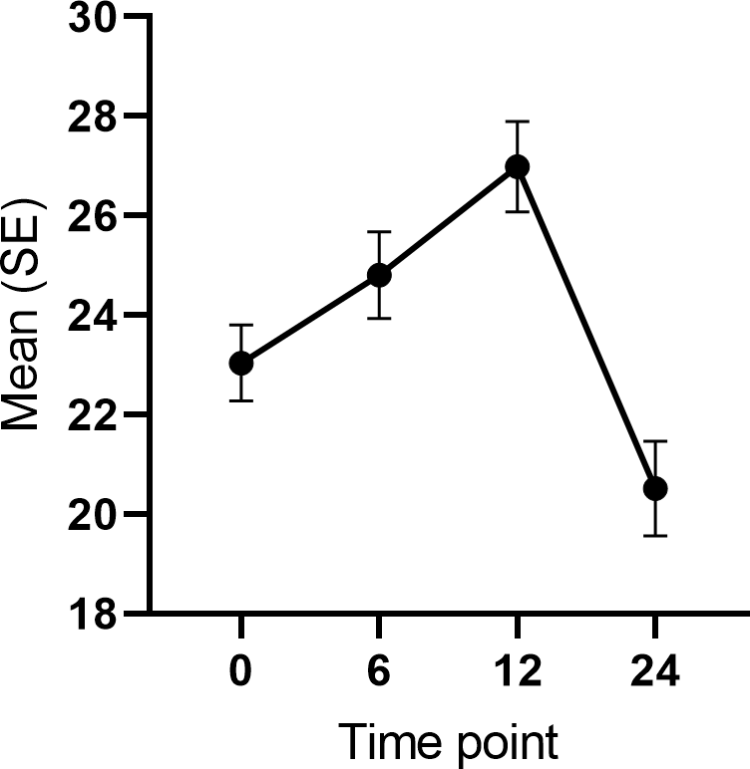
Changes in eating competence in all participants during the 12-month intervention and further 12-month follow-up without additional support.

**Figure 5.**
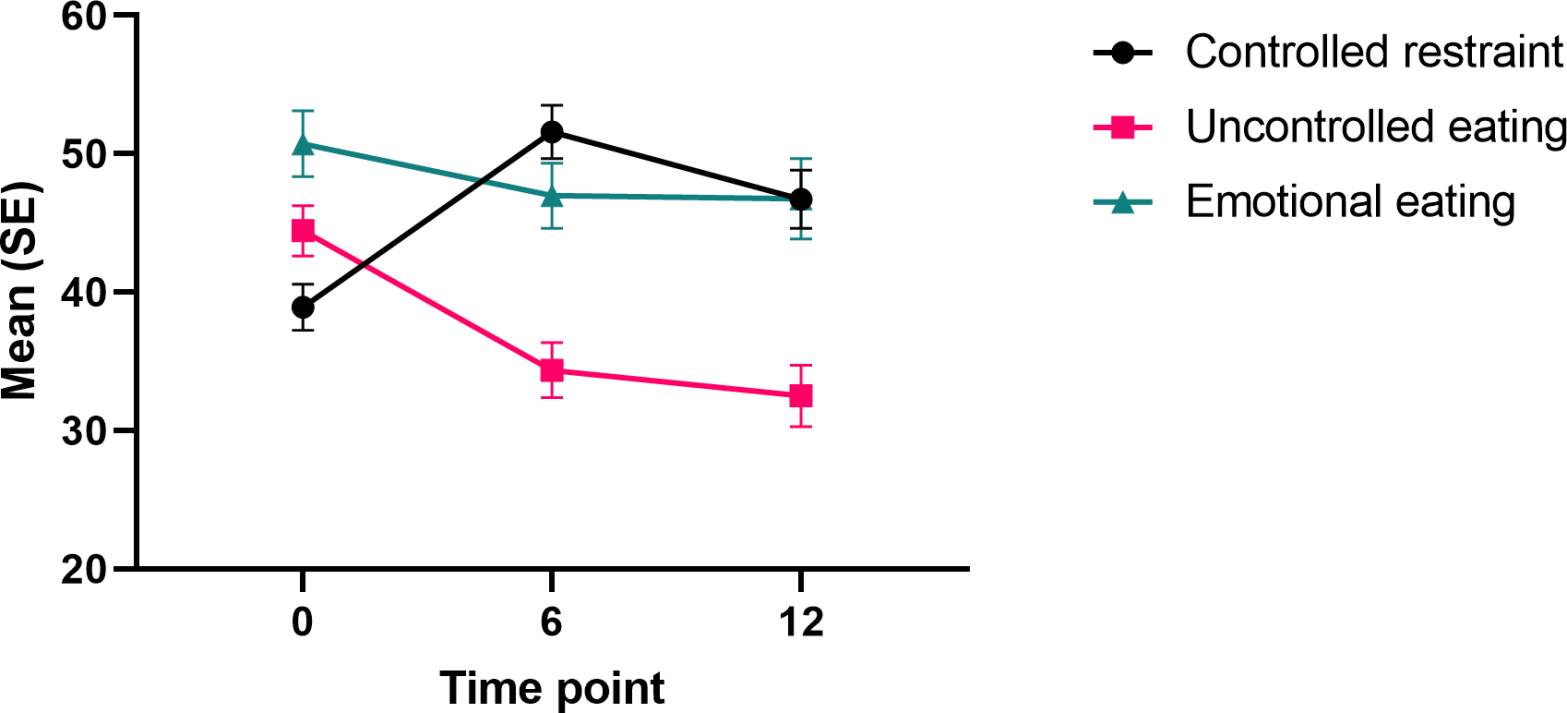
Changes in controlled restraint, uncontrolled eating, and emotional eating in all participants during the 12-month intervention.

Specifically, eating competence increased between 0- and 6-month (*χ*^2^_1_=5.34; *P*=.02; *d*=0.23) and 0- and 12-month time points (*χ*^2^_1_=22.86; *P<*.001; *d*=0.53), and decreased between 0- and 24-month time points (*χ*^2^_1_=7.27; *P*<.01; *d*=−0.36; Figure 4). The subscale analysis revealed increases in Contextual skills at 0‐6 months (*χ*^2^_1_=25.35; *P*<.001; *d*=0.52) and 0‐12 months (*χ*^2^_1_=37.09; *P*<.001; *d*=0.69) and Food acceptance in 0‐12 months (*χ*^2^_1_=37.61; *P*<.001; *d*=0.92), and a decrease in the Eating attitudes subscale at 0‐24 months (*χ*^2^_1_= ; *P*<.001; *d*=−0.68; Figure 5). The change in eating competence was not explained by any of the controlled variables when using the full covariate model.

Uncontrolled eating decreased between the 0‐6-month (*χ*^2^_1_=43.29; *P<*.001; *d*=0.54) and 0‐12–month time points (*χ*^2^_1_=25.90; *P<.*001; *d*=0.67). The decrease in uncontrolled eating was explained by age (*χ*^2^_2_=7.97; *P=*.02) and 6-month weight change (*χ*^2^_2_=8.44; *P*=.02), with older participants and those who lost more weight showing greater reduction in uncontrolled eating (Table S7 in Multimedia Appendix 1). The main effect of time on uncontrolled eating did not remain significant (*χ*^2^_2_=2.69; *P*=.26*)* after controlling for age and weight change, indicating that the observed changes were attributed to these confounders.

Conversely, controlled restraint increased from baseline to 6 months (*χ*^2^_1_=44.87; *P<*.001; *d*=0.74) and to 12-months (*χ*^2^_1_=14.20; *P*<.001; *d*=0.40; Figure 5). Covariate analysis (Table S7 in Multimedia Appendix 1) revealed that the increase in controlled restraint was greater in the participants who lost more weight from baseline to 6 months (*χ*^2^_2_=15.92; *P*<.001) and to 12 months (*χ*^2^_2_=27.90; *P*<.001). The main effect of time on controlled restraint remained significant (*χ*^2^_2_=30.46; *P*<.001 and *χ*^2^_2_=30.64; *P*<.001, respectively) after controlling for changes in weight.

**Figure 6.**
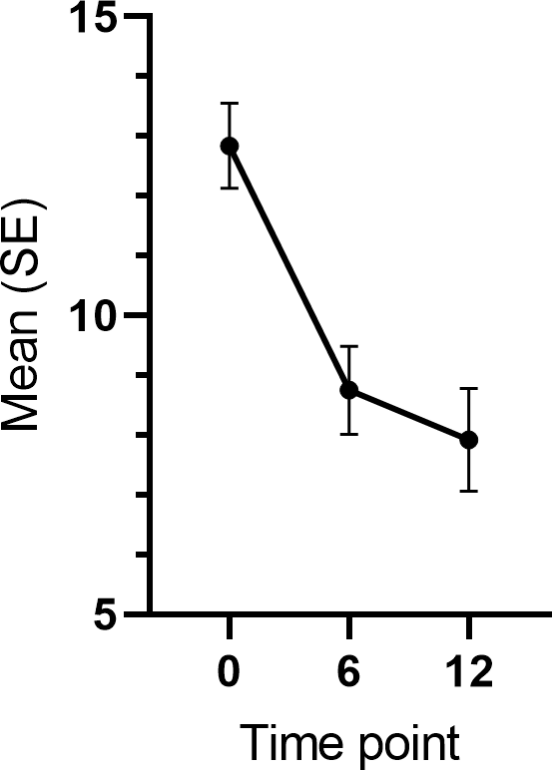
Changes in binge eating tendency during the 12-month intervention in all participants.

Binge eating tendency showed a steady decline, decreasing 4.9 BES-points during the intervention (0‐6 month *χ*^2^_1_=31.51; *P*<.001; *d*=0.58 and 0‐12 month *χ*^2^_1_=22.97; *P*<.001; *d*=0.67 Figure 6). The covariate analysis revealed that the participants who lost more weight from baseline to 6 months exhibited greater improvement in the binge eating tendency (*χ*^2^_2_=4.88; *P*=.03). The main effect of time remained significant in the full covariate model (*χ*^2^_2_=42.14; *P*<.001).

Physical activity (*χ*^2^_1_=13.51; *P*<.001; *d*=0.38), and the sports subscale (*χ*^2^_1_=18.54; *P*<.001; *d*=0.44) increased between 0- and 12-month time points. The change in physical activity was not explained by any of the controlled variables.

## Discussion

### Principal Findings

This study examined whether minimal group or individual enhancement would offer additional benefits to a 12-month eHealth weight maintenance intervention on mental health, perceived work ability, and health behavior. We observed the 3 intervention arms to change differently over time in terms of the depression and burnout scores. Nevertheless, post hoc analysis revealed that these changes were not consistent over the 12- and 24-month follow-up periods, indicating fluctuations rather than meaningful intervention effects. Furthermore, the different trajectories of the treatment arms were explained by baseline use and changes in psychotropic medication for both depression and burnout. In addition, 12-month weight change and sex contributed to the differences in depression and burnout, respectively. These results further indicated that the observed group-level differences were explained by factors other than the intervention.

In addition, we sought to explore the overall effects of an ACT-based weight management intervention on these domains. Across the entire study population, we observed statistically significant reductions in depressive symptoms and binge eating tendencies, along with improvements in eating competence and physical activity. Although the overall findings were quasi-experimental and must be interpreted cautiously, they collectively suggest that the standard HWC protocol may provide mental health and health behavioral benefits.

### Comparison With Previous Work

While hybrid weight management interventions have shown superiority over digital interventions in somatic health, no relevant studies have reported their differences in mental health domains. In mental health interventions, personal guidance has been shown to enhance efficacy compared with unguided approaches [51]. Regarding health behavior, early findings suggest that personalized coaching leads to greater reductions in emotional eating than fully digital weight maintenance programs [52]. To our knowledge, no previous research has directly compared coach-assisted eHealth interventions with those supplemented by face-to-face contact. Given the low engagement levels of stand-alone digital applications, theoretical considerations suggest incorporating face-to-face elements to improve the efficacy and acceptability of interventions [30,53,54].

In our study design, each participant was supported by the same coach through written communication in the eHealth program, and during all face-to-face sessions in the enhanced arm. Therefore, we hypothesized that group or individual meetings would strengthen the therapeutic alliance [55] with the coach, leading to greater engagement with the program, increased exposure to therapeutic content, and, ultimately, improved outcomes.

Several factors may explain the absence of meaningful intergroup differences. One possibility is that the 3 sessions in the hybrid conditions were insufficient to create a meaningful distinction from the standard HWC intervention in relation to mental health variables, mirroring the findings of our previous paper on somatic health [31]. Increasing the number of sessions in the hybrid intervention might have led to more noticeable differences between the treatment arms. Alternatively, written coach assistance alone may provide sufficient support to participants, rendering face-to-face sessions unnecessary.

To our knowledge, no previous studies have examined the effects of an ACT-based eHealth weight loss intervention on depression. While our results with the overall sample are preliminary due to the quasi-experimental design, the small-to-medium effect size reduction in depressive symptoms across the entire sample is consistent with previous research demonstrating the effectiveness of ACT interventions for depression [56,57], including in the context of eHealth [58] and weight loss interventions [21]. Although our study included a general population that on average did not score within the range of clinical depression, the result might be meaningful from a population health perspective. Notably, among the completers, a reduction in the number of participants with clinical depression was observed. This effect was not evident in the intention-to-treat analysis, which may indicate the importance of retention in meaningful mental health improvements. Furthermore, while eHealth interventions targeting weight loss have been shown to effectively reduce depression symptoms in participants with obesity [59], our results show that the reduction in depressive symptoms was not explained by the amount of weight loss. This is supported by a meta-analysis by Fabricatore et al [60], which showed that nondiet interventions can significantly reduce depression symptoms in the absence of weight loss.

Contrary to expectations, we did not observe changes in burnout symptoms or perceived work ability between the baseline and postintervention assessments. Interestingly, burnout symptoms increased between the baseline and the 6-month time point, which contradicts previous research showing that ACT-based interventions can alleviate severe burnout [57,61]. Given the strong influence of workplace psychosocial factors on work ability [5], these findings suggest that interventions focused solely on individual change may be insufficient to impact occupational well-being. Future efforts might benefit from incorporating workplace-focused interventions along with individual treatments to enhance work ability more effectively.

Our results show positive changes in eating behavior, with medium-to-large effect sizes, and physical activity, with small to medium effect sizes. These results align with previous studies reporting positive effects of ACT-based interventions on eating behavior [62-65] and physical activity [65,66].

Overall eating competence improved during the intervention, particularly in the food acceptance and eating context subdomains, suggesting a more versatile, mindful, and planned approach to eating. Despite these improvements, eating competence was not sustained at the follow-up, as indicated by a significant decline in the eating attitude subscore, which reflects positive emotions related to eating. This suggests that, following the end of the intervention, the participants became less relaxed and comfortable about eating. The weight-neutral approach may have initially promoted a more balanced attitude toward eating, but once the intervention ended, participants may have struggled to maintain this mindset independently. Considering that longer treatment durations are associated with more substantial improvements in dysregulated eating behaviors [64], providing continued support during the follow-up phase could help preserve any positive changes achieved during the intervention.

The binge eating tendency decreased with medium-to-large effect sizes, consistent with previous ACT research [64,65,67]. Unlike behavioral interventions that emphasize daily self-weighing, an approach that has not been shown to impact binge eating [68], our intervention integrated ACT and behavioral strategies without imposing external constraints, such as calorie restrictions or mandatory physical activity plans. This suggests that a more intuitive treatment approach might be more effective in reducing binge eating than focusing on weight loss. Furthermore, we observed an increase in controlled restraint, indicating that the participants’ conscious efforts to restrict their food intake increased during the intervention. This finding contrasts with previous ACT-based weight loss interventions, which did not report significant increases in controlled restraint [65,67]. This result appears to contradict the “restraint theory,” which suggests that higher controlled restraint may lead to higher levels of overeating and binge eating [69]. Nevertheless, this effect may differ in populations with obesity, where increased controlled restraint has been linked to a decreased binge eating tendency [70]. This discrepancy could also be attributed to the method used in this study. The 18-item version of the TFEQ used here does not distinguish between rigid and flexible cognitive restraint, with the latter being associated with successful weight management [71].

### Strengths and Limitations

The strengths of this study include the use of an RCT setting to explore the differences between treatment arms. Furthermore, we used validated questionnaires with robust psychometric properties and a lengthy follow-up period of up to 24 months for some measures, providing a comprehensive view of the long-term effects.

Despite these strengths, there are several limitations to consider. First, the requirement for internet access and the predominantly female, middle-aged, and “white-collar” sample limits generalizability to other populations. Although we controlled for sex and age using them in stratification and including them as covariates in our analyses, these characteristics are still likely to influence the outcomes. Second, the analysis could have been underpowered, as we used prospective weight change rather than mental health outcomes to calculate the statistical power, which may have led to biases in the observed effect sizes. Third, the substantial amount of missing data due to attrition and inactivity may have affected the reliability of the questionnaires used in this study. To mitigate potential bias from missing data, we used a statistical method with an intention-to-treat approach and applied the necessary statistical corrections. Fourth, although the between-group analysis was conducted in an RCT setting, the overall analysis relied on a quasi-experimental design. We were therefore unable to fully evaluate the individual intervention effect without a control group for comparison, making the overall results sensitive to bias.

Finally, the timing of the intervention during the COVID-19 pandemic may have substantially affected the results. The pandemic’s social restrictions, along with the increase in remote work, could have exacerbated the symptoms of burnout and disordered eating, as well as reduced physical activity levels. These factors may have confounded the observed effects of the intervention. In addition, the need to adapt the intervention to a remote format, which had originally been planned for in-person support groups, may have further affected the effectiveness of the support provided.

### Conclusions

Minimal enhancement through either group or individual video-conference meetings did not provide additional benefits in the mental health or eating habit domains compared with the eHealth intervention alone. Across the entire study population, we observed positive changes in depressive symptoms, binge eating tendencies, eating competence, and physical activity during the intervention. Future research should continue to explore the differences between hybrid and digital interventions on mental health domains, distinguishing for delivery mode and contact frequency, as well as the impact of continued support for the maintenance of positive outcomes.

## Supplementary material

10.2196/66518 Multimedia Appendix 1 Supplementary tables.

10.2196/66518 Checklist 1 CONSORT-eHEALTH checklist (V 1.6.1).

## Abbreviations

ACT: acceptance and commitment therapy
BDI-21: Beck Depression Index 21
BES: Binge Eating Scale
BHPAQ: Baecke Habitual Physical Activity Questionnaire
HWC: health weight coaching
MCAR: missing completely at random
RCT: randomized controlled trial
RCT: randomized controlled trial
TFEQ: Three Factor Eating Questionnaire
