# Supplementary material for: Effect of Minimal Individual or Group Enhancement in an eHealth Program on Mental Health, Health Behavior, and Work Ability in Employees With Obesity: Randomized Controlled Trial
Source: JMIR Ment Health. 2025 Jul 7;12:e66518. doi: 10.2196/66518 (PMC12304238; doi:10.2196/66518)
Supplement: Multimedia Appendix 1 [file mental-v12-e66518-s001.docx]

| Table S1. Central themes of the Healthy Weight Coaching program, incorporating behavioral weight management and acceptance and commitment therapy (ACT). | | |
| --- | --- | --- |
|  | **Theme** | **E.g. subthemes and exercises** |
| **Behavioral weight management** | Eating habits | Meal rhythm diary, picture food diary  Plan for adding vegetables, the role of sugar in the diet  Hunger scale |
|  | Physical activity | Choose tips for increasing everyday movement  Neighborhood walk  Mini-exercises |
|  | Sleep | Factors affecting sleep and examining one's own sleep habits  The effect of alcohol on sleep  Sleep-promoting movement |
|  | Stress management | Stress management techniques and personal ways to regulate stress  Give yourself relaxation moments  The effect of stress on bodily functions |
| **ACT processes** | Values | Things important to you |
|  | Committed action | Everyday goals and actions  Actions that fit your life situation and values |
|  | Contact with the present moment | Following your breath  Body scan  Mindful eating |
|  | Acceptance | Self-compassion and acceptance in the present moment (thoughts, feelings, bodily sensations, pace of change) |
|  | Defusion | Important actions even with unpleasant emotions  Exposure to temptations  Internal dialogue and thoughts sabotaging change |
|  | Self as context | Lifestyle changes through self-esteem  Weight and body image  My strengths |

Table S2. The agenda of the group meetings in the eHealth+Group treatment arm.

| **Meeting** | **Theme** | **Psychoeducation** | **Exercise** | **Pair/group discussion** |
| --- | --- | --- | --- | --- |
| 1 | Flexibility in weight loss | The paradox of weight loss, flexible restraint in eating and exercising | “Don’t think about the jelly-filled donuts” | Lessons learned from previous weight loss attempts  Where could I introduce more flexibility? Where do I feel flexibility is already achievable? |
| 2 | Stress regulation and mindfulness | The impact of stress-management in weight loss, mindfulness, consistency and moderation | Mindful breathing | What restores you and how is it part of your daily life? Experiences with mindfulness? |
| 3 | Self-compassion and acceptance | Acceptance and self-compassion in lifestyle change. changing body | Difficult moment, the observing self | What are you grateful for in your body today?  Realizations over the intervention, what would you like to carry forward into the future? |

Figure S1. Flow of the participants through the three study groups (eHealth, eHealth+Group and eHealth+Individual) in the randomized controlled trial, showing the variation in the number of respondents across the questionnaires.

*
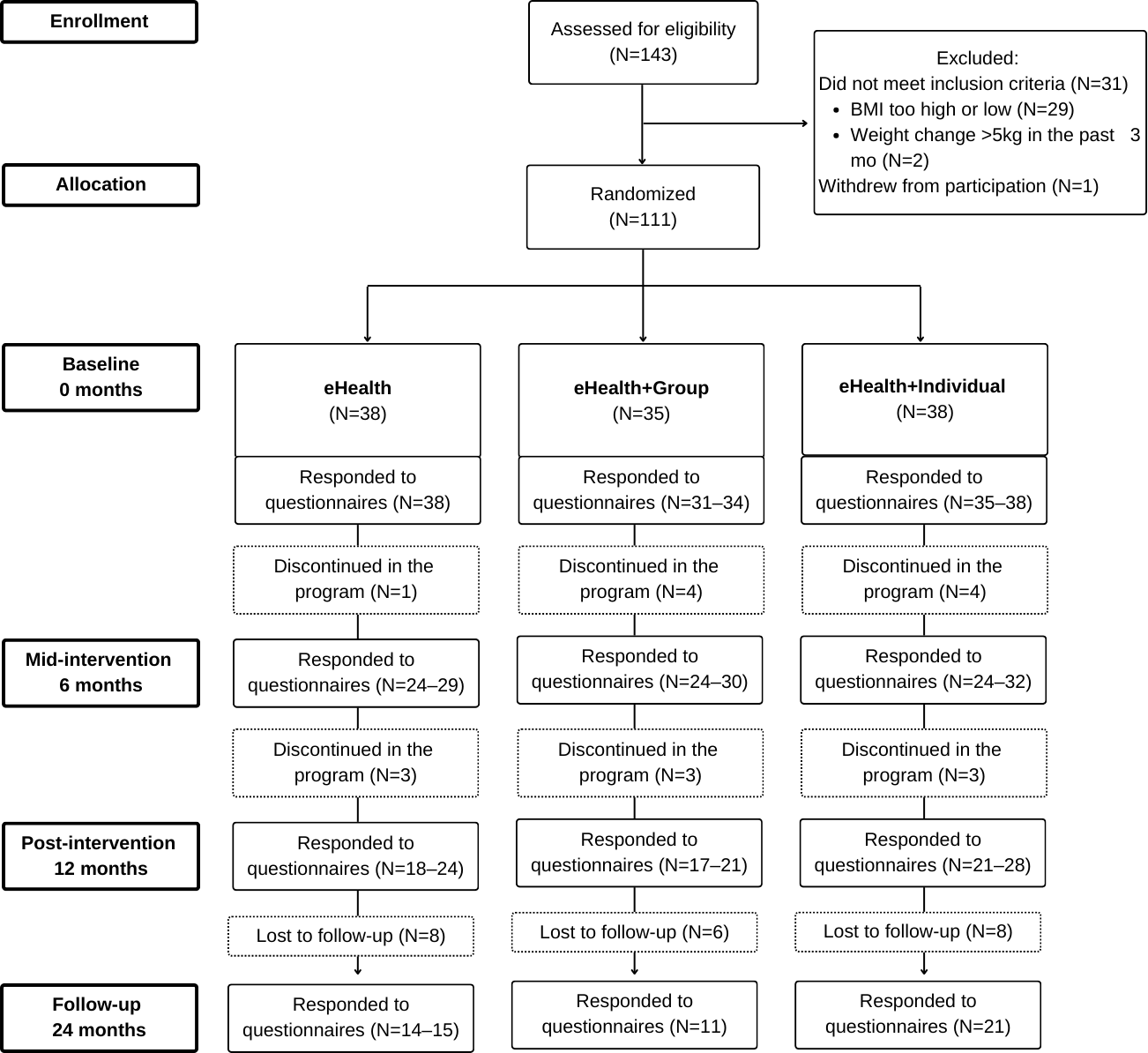
*

| Table S3. Psychotropic medication usage at the baseline and changes during the 12-month intervention and subsequent 12-month follow-up, categorized by the type of change (decreased, discontinued, increased and initiated). | | | | |
| --- | --- | --- | --- | --- |
|  | eHealth | eHealth+Group | eHealth+Individual | **Total** |
| **Baseline** | 6 | 2 | 7 | 15 |
| **Mid-intervention changes** | |  |  | 3 |
| Decreased |  |  | 1 | 1 |
| Discontinued |  |  |  | 0 |
| Increased |  |  |  | 0 |
| Initiated | 1 | 1 |  | 2 |
| **Follow-up** |  |  |  | 4 |
| Decreased |  |  |  | 0 |
| Discontinued |  | 1 | 1 | 2 |
| Increased |  |  |  | 0 |
| Initiated |  | 1 | 1 | 2 |

| Table S4. Baseline characteristics by treatment group with significance of the between-group differences analyzed by Fisher Exact for the categorized variables and ANOVA or Kruskal-Wallis test for the continuous variables, based on their parametric. | | | | | | | | | | | | | | | | | | | | | | | | |
| --- | --- | --- | --- | --- | --- | --- | --- | --- | --- | --- | --- | --- | --- | --- | --- | --- | --- | --- | --- | --- | --- | --- | --- | --- |
| **Characteristic** | | | **Treatment arm** | | | | | | | | | | | | | | | | |  | | | | |
|  | | | **eHealth**  **(N=38)** | **eHealth+**  **Group**  **(N=34)** | | | | | **eHealth+**  **Individual (N=38)** | | | | **Total**  **(N=110)** | | | | | ***P*value** | | |  |  |  |  |
| **Age (years)^1^** | | | **52.3 (9.7)** | **50.6 (7.9)** | | | | | **49.6 (8.8)** | | | | **50.0 (8.9)** | | | | | **.41** | | |  |  |  |  |
| **Sex, n (%)^1^** | | |  |  | | | | |  | | | |  | | | | | **.53** | | |  |  |  |  |
| Male | | | 8 (21%) | 4 (11%) | | | | | 7 (18%) | | | | 19 (17%) | | | | |  | | |  |  |  |  |
| Female | | | 30 (79%) | 31 (89%) | | | | | 31 (82%) | | | | 92 (83%) | | | | |  | | |  |  |  |  |
| **Professional role^1^** | | |  |  | | | | |  | | | |  | | | | | **.79** | | |  |  |  |  |
| Managers | | | 0 (0%) | 2 (6%) | | | | | 2 (5%) | | | | 4 (4%) | | | | |  | | |  |  |  |  |
| Specialized Experts | | | 15 (40%) | 9 (26%) | | | | | 10 (26%) | | | | 34 (31%) | | | | |  | | |  |  |  |  |
| Experts | | | 14 (37%) | 17 (49%) | | | | | 15 (39%) | | | | 46 (41%) | | | | |  | | |  |  |  |  |
| Office and Customer Service Workers | | | 4 (11%) | 4 (11%) | | | | | 5 (13%) | | | | 13 (12%) | | | | |  | | |  |  |  |  |
| Service and Sales Workers | | | 5 (13%) | 2 (6%) | | | | | 5 (13%) | | | | 12 (11%) | | | | |  | | |  |  |  |  |
| Construction, Repair, and Manufacturing Workers | | | 0 (0%) | 1 (3%) | | | | | 1 (3%) | | | | 2 (2%) | | | | |  | | |  |  |  |  |
| **Mental health & work ability** | | | | | | | | | | | | | | | | | | | | |  |  |  |  |
| Mental health problem**^1^** | | | 8 (21%) | 5 (14%) | | | | | 10 (26%) | | | | 23 (21%) | | | | | .45 | | |  |  |  |  |
| Depression | | | 10.1 (1.1) | 8.7 (1.1) | | | | | 11.5 (1.4) | | | | 10.1 (0.7) | | | | | .442 | | |  |  |  |  |
| Clinical depression | | | 12 (32%) | 8 (24%) | | | | | 16 (42%) | | | | 36 (33%) | | | | | .258 | | |  |  |  |  |
| Burnout | | | 40.8 (2.1) | 37.5 (2.4) | | | | | 40.5 (2.5) | | | | 39.7 (1.3) | | | | | .613 | | |  |  |  |  |
| Exhaustion^2^ | | | 14.5 (0.7) | 13.3 (0.8) | | | | | 13.7 (0.8) | | | | 13.9 (0.5) | | | | | .346 | | |  |  |  |  |
| Cynicism | | | 12.2 (0.8) | 11.4 (0.8) | | | | | 12.6 (0.9) | | | | 12.1 (0.5) | | | | | .666 | | |  |  |  |  |
| Efficacy | | | 14.2 (1.0) | 13.4 (1.0) | | | | | 14.2 (1.0) | | | | 13.9 (0.6) | | | | | .815 | | |  |  |  |  |
| Clinical burnout | | | 10 (26%) | 11 (32%) | | | | | 11 (29%) | | | | 32 (29%) | | | | | .837 | | |  |  |  |  |
| Perceived work ability | | | 8.0 (0.2) | 7.8 (0.2) | | | | | 7.8 (0.3) | | | | 7.9 (0.2) | | | | | .648 | | |  |  |  |  |
| Reduced work-ability | | | 10 (26%) | | | | 10 (29%) | | | | 16 (42%) | | | | | 36 (33%) | | .299 | | | | |  |  |
| **Eating behavior** | | |  |  | | | | |  | | | |  | | | | |  | | |  |  |  |  |
| Eating competence^3^ | | | 24.0 (1.3) | | 22.4 (1.2) | | | | | 23.1 (1.5) | | | | 23.2 (0.8) | | | | .709 | | | |  |  |  |
| Eating attitudes^4^ | | | 9.5 (0.5) | | 8.4 (0.6) | | | | | 8.6 (0.5) | | | | 8.8 (0.3) | | | | .377 | | | |  |  |  |
| Food acceptance^5^ | | | 3.7 (0.4) | | 3.3 (0.4) | | | | | 4.1 (0.4) | | | | 3.7 (0.2) | | | | .451 | | | |  |  |  |
| Regulation of food intake^5^ | | | 5.1 (0.4) | | 4.9 (0.4) | | | | | 4.7 (0.4) | | | | 4.9 (0.2) | | | | .821 | | | |  |  |  |
| Eating context^6^ | | | 5.8 (0.5) | | 5.6 (0.5) | | | | | 5.6 (0.6) | | | | 5.7 (0.3) | | | | .984 | | | |  |  |  |
| ‘Poor eating competence’^3^ | | 33 (87%) | | | 29 (94%) | | | | 29 (83%) | | | | | 91 (88%) | | | | .461 | | | | | |  |
| Controlled restrained^7^ | | | 43.2 (3.1) | | 37.7 (2.9) | | | | | 34.0 (2.5) | | | | 38.4 (1.7) | | | | .079 | | | |  |  |  |
| Uncontrolled eating^4^ | | | 43.4 (3.1) | | 45.0 (3.2) | | | | | 43.9 (3.4) | | | | 44.8 (1.8) | | | | .948 | | | |  |  |  |
| Emotional eating^8^ | | | 53.3 (4.4) | | 49.1 (4.9) | | | | | 49.6 (3.3) | | | | 50.9 (2.4) | | | | .798 | | | |  |  |  |
| Binge eating tendency^2^ | | | 13.2 (1.3) | | 12.4 (1.1) | | | | | 12.6 (1.3) | | | | 12.8 (0.7) | | | | .966 | | | |  |  |  |
| Moderate or severe binge eating tendency^2^ | | 7 (18%) | | | | | | 3 (9%) | | | | 6 (16%) | | | | | 16 (15%) | | .554 | | | | |  |

| **Physical activity**^2^ | 11.1 (0.2) | 10.6 (0.2) | 10.9 (0.3) | 10.9 (1.5) | .447 |
| --- | --- | --- | --- | --- | --- |
| Physical activity at work^2^ | 2.1 (0.1) | 2.1 (0.1) | 2.2 (0.1) | 2.1 (0.04) | .792 |
| Sports^2^ | 2.6 (0.2) | 2.6 (0.2) | 2.4 (0.2) | 2.6 (0.96) | .269 |
| Leisure time activity^2^ | 6.3 (0.2) | 5.9 (0.1) | 6.3 (0.2) | 6.2 (0.1) | .002 |

^1^ N=111, ^2^ N=109, ^3^ N=104, ^4^ N=108, ^5^ N=107, ^6^ N=105, ^7^ N= 100, ^8^ N=88

Table S5. Differences between baseline clinical study population and psychotropic medication users and the non-clinical population on baseline questionnaire scores, analyzed by Independent-Samples Kruskal-Wallis Test (2-sided).

| Independent variable | Dependent variable | Total N | H value (df=1) | *P* |
| --- | --- | --- | --- | --- |
| BDI score above cut-off | |  |  |  |
|  | Burnout score | 110 | 20.94 | <.001 |
|  | Perceived work ability score | 110 | 18.91 | <.001 |
|  | Eating competence score | 104 | .35 | .56 |
|  | Emotional eating score | 88 | 5.34 | .02 |
|  | Controlled restraint score | 100 | .03 | .86 |
|  | Uncontrolled eating | 108 | 1.77 | .18 |
|  | Binge eating score | 109 | 11.35 | <.001 |
|  | Physical activity score | 109 | 1.07 | .30 |
| BBI score above cut-off | |  |  |  |
|  | Depression score | 110 | 19.35 | <.001 |
|  | Perceived work ability score | 110 | 10.98 | <.001 |
|  | Eating competence score | 104 | 4.93 | .03 |
|  | Emotional eating score | 88 | 3.23 | .07 |
|  | Controlled restraint score | 100 | .12 | .73 |
|  | Uncontrolled eating | 108 | 2.86 | .09 |
|  | Binge eating score | 109 | 3.85 | .050 |
|  | Physical activity score | 110 | 1.61 | .21 |
| Psychotropic medication | |  |  |  |
|  | Depression score | 110 | 8.59 | .003 |
|  | Burnout score | 110 | 2.86 | .09 |
|  | Perceived work ability score | 110 | 10.77 | .001 |
|  | Eating competence score | 104 | 3.23 | .07 |
|  | Emotional eating score | 88 | .53 | .47 |
|  | Controlled restraint score | 100 | 4.63 | .03 |
|  | Uncontrolled eating | 108 | 6.06 | .01 |
|  | Binge eating score | 109 | 10.83 | <.001 |
|  | Physical activity score | 109 | .50 | .48 |

Table S6. Completers' mean (SE) changes from baseline to 6 and 12 or 24 months by treatment arm (eHealth, eHealth+Group, eHealth+Individual), statistical analysis conducted using the Generalized Estimating Equations with the input of completer data.

|  | **0 to 6 mo change** | | | **0 to 12 mo change** | | | **0 to 24 mo change** | | |  |  | |
| --- | --- | --- | --- | --- | --- | --- | --- | --- | --- | --- | --- | --- |
|  | **eHealth**  **(N=29)** | **eHealth+**  **Group**  **(N=30)** | **eHealth+**  **Indiv.**  **(N=31)** | **eHealth**  **(N=18)** | **eHealth+**  **Group**  **(N=18)** | **eHealth+**  **Indiv.**  **(N=23)** | **eHealth**  **(N=14)** | **eHealth+**  **Group (N=11)** | **eHealth+**  **Indiv.**  **(N=21)** | **Wald chi^2^** | ***P*^^^** |  |
| Depression | -0.18 (0.75) | 0.55 (0.68) | -3.05 (0.89) | -3.77 (1.06) | -1.44 (0.83) | -2.51 (1.27) |  |  |  | 17.19 | .002 |  |
| Burnout | 5.40 (2.77) | 7.34 (2.23) | -0.09 (1.59) | 1.24 (2.24) | -1.02 (2.71) | 1.78 (1.94) | 1.04 (2.99) | 5.62 (3.95) | 2.30 (2.75) | 12.91 | .044 |  |
| Exhaustion | 1.00 (0.81) | 2.18 (0.84) | 0.27 (0.71) | -0.78 (0.66) | 0.16 (0.85) | 0.18 (0.86) | -0.44 (1.18) | 1.70 (1.00) | 0.95 (0.96) | 6.80 | .34 |  |
| Cynicism | 2.64 (1.06) | 2.84 (0.77) | -0.24 (0.56) | 0.82 (1.03) | 0.19 (0.99) | 0.57 (0.80) | 0.49 (0.99) | 2.29 (1.64) | 0.49 (0.94) | 17.01 | .009 |  |
| Efficacy | 1.77 (1.12) | 2.21 (0.88) | -0.14 (0.62) | 1.20 (1.04) | -1.64 (1.21) | 1.02 (0.72) | 0.86 (1.29) | 1.30 (2.06) | 0.83 (1.12) | 10.83 | 0.09 |  |
| Perceived work ability | -0.44 (0.31) | -0.14 (0.26) | -0.49 (0.28) | 0.29 (0.24) | -0.19 (0.27) | -0.55 (0.33) | -0.31 (0.48) | -0.18 (0.57) | -0.12 (0.24) | 9.02 | .72 |  |
| Eating competence | 1.57 (0.13) | 1.04 (1.02) | 2.74 (1.60) | 3.36 (1.41) | 4.70 (1.44) | 3.98 (1.42) | -0.91 (1.54) | -2.41 (1.48) | -3.67 (1.63) | 6.46 | .37 |  |
| Controlled restaint | 10.04 (3.38) | 9.52 (2.81) | 17.39 (3.04) | 7.61 (4.00) | 4.66 (3.19) | 10.98 (3.21) |  |  |  | 5.59 | .23 |  |
| Uncontrolled eating | -9.00 (2.25) | -11.65 (2.52) | -9.57 (2.41) | -12.81 (3.20) | -16.20 (3.76) | -8.12 (3.68) |  |  |  | 2.91 | .57 |  |
| Emotional eating | -5.55 (4.34) | -4.49 (4.82) | -0.83 (3.05) | -12.07 (5.92) | -0.97 (6.04) | 0.63 (5.78) |  |  |  | 3.77 | .44 |  |
| Binge eating tendency | -4.48 (0.89) | -4.54 (0.98) | -3.53 (1.21) | -6.78 (1.25) | -6.72 (1.77) | -4.64 (1.42) |  |  |  | 1.74 | .78 |  |
| Physical activity | 0.00 (0.19) | 0.29 (0.18) | 0.39 (0.18) | 0.50 (0.24) | 0.41 (0.19) | 0.63 (0.27) |  |  |  | 5.29 | .26 |  |

^^^*P* value indicating significance of the between-group change across the four measurement points (0-6-12-24 mo)

Table S7. Preliminary covariate model showing the effect of age, sex, weight change, psychotropic medication, and medication usage on significant changes in the main variable (rows) from the single-group analysis, analyzed using GEE with covariates (columns) added to the model individually.

| Measurements | Age |  | Sex |  | Weight change % 0-6 mo | | Weight change % 0-12 mo | | Psychotropic medication usage | | Psych. medication change 0-12 mo | | Psych. medication change 12-24mo | |
| --- | --- | --- | --- | --- | --- | --- | --- | --- | --- | --- | --- | --- | --- | --- |
|  | Wald chi-square (df=3) | *P* | Wald chi-square (df=3) | *P*^ | Wald chi-square (df=3) | *P* | Wald chi-square (df=3) | *P* | Wald chi-square (df=4) | *P* | Wald chi-square (df=4) | *P* | Wald chi-square (df=4) | *P* |
| Depression | 14.73 | <.001 | 1.37 | .51 | 9.90 | .007 | 9.54 | .008 | .55 | .76 | 3.14 | .21 |  |  |
| Burnout | 3.82 | .28 | 1.31 | .73 | 1.28 | .73 | .28 | .97 | 6.03 | .11 | 20.08 | <.001 | 6.04 | .11 |
| Exhaustion | 1.44 | .70 | 1.47 | .69 | 2.84 | .42 | .53 | .91 | 20.45 | <.001 | 78.00 | <.001 | 19.83 | <.001 |
| Cynicism | 3.40 | .33 | 2.93 | .40 | .78 | .85 | 1.40 | .71 | 1.85 | .60 | 20.27 | <.001 | 14.17 | .003 |
| Eating competence | 10.42 | .015 | 8.43 | .04 | 21.27 | <.001 | 12.22 | .007 |  |  |  |  |  |  |
| Eating attitudes | 9.01 | .03 | 1.51 | .68 | 6.06 | .11 | 2.27 | .52 |  |  |  |  |  |  |
| Food acceptance | 2.80 | .42 | 1.54 | .67 | 12.81 | .005 | 6.83 | .08 |  |  |  |  |  |  |
| Regulation of food intake | 1.42 | .70 | 9.97 | .02 | 4.32 | .23 | .46 | .93 |  |  |  |  |  |  |
| Eating context | 1.15 | .76 | 12.83 | .005 | 8.72 | .03 | 4.41 | .22 |  |  |  |  |  |  |
| Controlled restraint | 3.88 | .14 | .84 | .66 | 15.92 | <.001 | 27.90 | <.001 |  |  |  |  |  |  |
| Uncontrolled eating | 7.97 | .02 | .46 | .80 | 8.44 | .02 | 1.34 | .51 |  |  |  |  |  |  |
| Binge eating tendency | 10.53 | .005 | .49 | .78 | 11.06 | .004 | 3.82 | .15 |  |  |  |  |  |  |
| Physical activity | 0.32 | .85 | 2.58 | .28 | .77 | .68 | 4.03 | .13 |  |  |  |  |  |  |
| Work | 0.11 | .95 | 3.92 | .14 | 2.71 | .26 | 2.92 | .23 |  |  |  |  |  |  |
| Sports | 1.09 | .58 | 1.40 | .50 | 1.80 | .41 | 4.69 | .10 |  |  |  |  |  |  |
| Leisure | 1.55 | .46 | 1.66 | .44 | 2.86 | .24 | .53 | .77 |  |  |  |  |  |  |

| Table S8a. Categorical change in clinical depression incidence in the completers during the 12-month intervention. | | | |
| --- | --- | --- | --- |
|  | Clinical depression | No clinical depression | Total N |
| Baseline | 36 | 74 | 110 |
| 12 months | 9 | 49 | 59 |
|  |  |  | *P*=.02 |

| Table S8b. Categorical change in clinical depression incidence during the 12-month intervention with last observation carried forward. | | | |
| --- | --- | --- | --- |
|  | Clinical depression | No clinical depression | Total N |
| Baseline | 36 | 74 | 110 |
| 12 months | 27 | 83 | 110 |
|  |  |  | *P*=.23 |

| Table S9a. Categorical change in clinical burnout incidence in the completers during the first 6 months of the 12-month intervention. | | | |
| --- | --- | --- | --- |
|  | Clinical burnout | No clinical burnout | Total N |
| Baseline | 32 | 78 | 110 |
| 6 months | 37 | 54 | 91 |
|  |  |  | *P*=.10 |

| Table S9b. Categorical change in clinical burnout incidence during the 6 months of the intervention with last observation carried forward. | | | |
| --- | --- | --- | --- |
|  | Clinical burnout | No clinical burnout | Total N |
| Baseline | 32 | 78 | 110 |
| 6 months | 43 | 67 | 110 |
|  |  |  | *P*=.16 |
